# Prescriptions and Dispensing

**Published:** 1908-01-11

**Authors:** 


### THERAPEUTICS.

### PRESCRIPTIONS AND DISPENSING.

Perchloride of iron is sometimes prescribed inadvertently with potassium iodide; such a mixture develops free iodine on standing, the iron being reduced to the ferrous state. The darkening of the medicine may alarm the patient, and the amount of iodine liberated may reach a dangerous degree. In the following prescription this reaction is complicated by secondary ones:

℞ Ferri et Quininæ Citratis ... ... ʒij.

Potassii Iodidi ... ... ... ... ʒiij.

Syrupi ... ... ... ... ... ʒvi.

Aquam ... ... ... ... ad ℥iv.

The amount of acid here, in the solution of the double citrate, is small, and iodine is only slowly liberated; iodine in potassium iodide solution is a general precipitant of alkaloids, and a nearly black precipitate of periodide of quinine will be produced. Addition of enough alkali to neutralise the solution of the scale salt before adding it to the potassium iodide will delay the reaction considerably.

℞ Quininæ Sulphatis ... ... ... ... gr. xv.

Acidi Sulphurici diluti ... ... ... ʒj.

Extracti Glycyrrhizæ liquidi ... ... ℥ss.

Aquam ... ... ... ... ad ℥iij.

The liquid extract of liquorice would be intended to cover the taste of the quinine, besides acting as a laxative, but the acid precipitates the glycyrrhizin, making a turbid mixture, and much reducing the sweetness of the liquorice. The better plan would be to omit the acid, and to suspend the quinine in mucilage instead.

Iodine may be liberated from an iodide in dangerous quantity by spirit of nitrous ether. Ordinary specimens of the latter are always acid, and if the acid is first neutralised by shaking the spirit with sodium bicarbonate, the reaction is much retarded. Caustic alkali must never be used in neutralising the spirit, as it would decompose the ethyl nitrite.

When antipyrine and spirit of nitrous ether are dispensed together in a mixture, reaction occurs, and a green liquid results. This reaction also is much retarded if the spirit is first neutralised, and this should always be done.

Another substance that is incompatible with this spirit is tannin. Ordinary tannic acid is not very likely to be prescribed with it, but very many vegetable drugs contain tannin, and some of these are occasionally the cause of trouble in such a mixture. The reaction that occurs involves decomposition of the ethyl nitrite, with production of nitric oxide; in a dilute liquid this only takes place slowly, and if the dispenser is not on his guard, the bottle may be burst. The medicine, if it is really wanted to be in that form, should be kept back as long as possible before delivery, in order that the reaction may be completed, and the patient should be warned to loosen the cork of the bottle as soon as it is received.

### Emulsions.

We now come to the special class of mixtures known as emulsions, in which two materially immiscible substances, such as oil and water, are brought into intimate mixture by the addition of a third substance, aided by proper manipulation. There is, of course, no hard and fast line between emulsions and some of the cases which we have already dealt with. For instance, the suspension of balsam of tolu in water by adding mucilage in a mixture containing tincture of tolu. An emulsion is understood to be a mixture of an oil, a resin, an oleoresin, or a greasy substance not strictly an oil, such as vaseline, with water or some aqueous vehicle.

If a little oil and water be shaken up together in a bottle, some degree of mixing does occur; the oil is broken up into globules of varying size, and these are distributed through the water; but as soon as shaking is stopped, the oil rises to the top, the globules coalesce again, and we have merely a layer of oil above a layer of water. In order to make a permanent mixture two things are necessary; first, some means by which the globules can be divided so minutely that the ultimate globules no longer appear as individuals; and secondly, some means of preventing the globules so formed from coalescing again. Coalescence can only occur when the globules come again into actual contact with one another, and in order that they should do this the vehicle which separates them must be one that does not cling at all to their surface. Water is such a liquid; but a watery solution of certain gums clings tenaciously to the surface of the globules, and so long as ever so thin a film persists between adjacent globules, they can no more run together than if they were widely separated. As regards the former requisite, the division of the oil into small globules, if we once secure that the globules shall not run together as fast as formed, there are several good means of subdivision. The method which is usually the best is gentle trituration in a mortar; if a little oil and mucilage, syrup, or glycerin are rapidly but lightly stirred together in a mortar, keeping the pestle moving in the same direction all the time, it is easy to see that each globule of oil is drawn out into a long shape, then it divides across the middle, and each of the smaller globules resulting is in turn drawn out and divided. With syrup or glycerin the limit of this process is soon reached, as the globules are not prevented from again coalescing; with mucilage we can carry it much further. This subdivision is, of course, only possible in a thick medium; otherwise the globules would slip away from the pestle.

When an oil or resin is made into an emulsion, there is another advantage besides the production of a uniform mixture. It is usually the case that the extremely small particles of oil can be more readily assimilated by the patient's alimentary canal than if a quantity of the oil were administered in the undivided state. This is a well-known fact with cod-liver oil, and furnishes a *raison d'être* for the numerous emulsions of this oil that are on the market. The doctor, in dispensing an emulsion himself, should always aim at the most perfect sub-division possible; and this not only makes the best preparation from a therapeutic point of view, but also renders the mixture more permanent; for, other things being equal, the finer the sub-division the more stable the emulsion.

